# Natural Deep Eutectic Solvents (NADESs) for the Extraction of Bioactive Compounds from Quinoa (*Chenopodium quinoa* Willd.) Leaves: A Semi-Quantitative Analysis Using High Performance Thin-Layer Chromatography

**DOI:** 10.3390/molecules30122620

**Published:** 2025-06-17

**Authors:** Verónica Taco, Dennys Almachi, Pablo Bonilla, Ixchel Gijón-Arreortúa, Samira Benali, Jean-Marie Raquez, Pierre Duez, Amandine Nachtergael

**Affiliations:** 1 Faculty of Chemical Sciences, Central University of Ecuador (UCE), Av. Universitaria, Quito PC 170129, Ecuador; dpalmachi@uce.edu.ec (D.A.); pmbonilla@uce.edu.ec (P.B.); 2 Unit of Therapeutic Chemistry and Pharmacognosy, Faculty of Medicine, Pharmacy and Biomedical Sciences, University of Mons (UMONS), Avenue du Champ de Mars, 25, 7000 Mons, Belgium; pierre.duez@umons.ac.be (P.D.); amandine.nachtergael@umons.ac.be (A.N.); 3 Faculty of Chemical Engineering, Autonomous University of Yucatán (UADY), Periférico Norte Km. 33.5, Tablaje Catastral 13615, Colonia Chuburná de Hidalgo Inn, Mérida 97203, Yucatán, Mexico; ixchel.gijon@correo.uady.mx; 4 Center of Innovation and Research in Materials, University of Mons (UMONS), Avenue du Champ de Mars, 25, 7000 Mons, Belgium; samira.benali@umons.ac.be (S.B.); jean-marie.raquez@umons.ac.be (J.-M.R.)

**Keywords:** eco-extraction, natural deep eutectic solvents, Amaranthaceae, high-performance thin layer chromatography-bioautography, antioxidant activity

## Abstract

Natural deep eutectic solvents (NADESs) have emerged as a promising eco-friendly alternative to petrochemicals for extracting plant metabolites. Considering that the demand for sustainable “green” ingredients for industrial applications is growing, those solvents are purported to develop extracts with interesting phytochemical fingerprints and biological activities. Given the interest in flavonoids from *Chenopodium quinoa* Willd. leaves, an efficient “green” extraction method was developed by investigating eight NADESs with defined molar ratios, i.e., malic acid-choline chloride (chcl)-water (w) (1:1:2, N1), chcl-glucose-w (5:2:5, N2), proline-malic acid-w (1:1:3, N3), glucose-fructose-sucrose-w (1:1:1:11, N4), 1,2-propanediol-chcl-w (1:1:1, N5), lactic acid-glucose-w (5:1:3, N6), glycerol-chcl-w (2:1:1, N7), and xylitol-chcl-w (1:2:3, N8). Rheological measurements of all NADESs confirmed their pseudoplastic behaviors. To improve the extraction processes, differential scanning calorimetry (DSC) allowed us to determine the maximum amount of water that could be added to the most stable NADES (N1, N2, N3, and N4; 17.5%, 20%, 10%, and 10% *w*/*w*, respectively) to lower their viscosities without disturbing their eutectic environments. The phytochemical compositions of NADES extracts were analyzed using high-performance thin-layer chromatography (HPTLC), and their free radical scavenging and α-amylase inhibitory properties were assessed using HPTLC-bioautography. N2, diluted with 20% of water, and N7 presented the best potential for replacing methanol for an eco-friendly extraction of flavonoids, radical scavengers, and α-amylase inhibitors from quinoa leaves. Their biological properties, combined with a good understanding of both thermal behavior and viscosity, make the obtained quinoa leaf NADES extracts good candidates for direct incorporation in nutraceutical formulations.

## 1. Introduction

In an era where industrial growth has been exponential, the significant negative effects of industrial processes on the environment are manifest [1]. Green chemistry aims at developing sustainable processes and/or solvents. In this context, deep eutectic solvents (DESs) and natural deep eutectic solvents (NADESs) have emerged as a recent generation of green solvents, sometimes called “smart advanced solvents” [2,3]. Deep eutectic solvents (DESs) are typically composed of two components: a quaternary ammonium salt (e.g., choline chloride) and a hydrogen bond donor (HBD), such as urea, carboxylic acids, or polyols. The formation of DESs, characterized by a significant depression in melting point to near room temperature, results from the establishment of strong intermolecular hydrogen bonds and van der Waals and/or electrostatic interactions between the quaternary ammonium salt and the HBD [4,5].

When the components of DESs are primary metabolites such as amino acids, organic acids (malic, citric, lactic, or succinic acids), sugars, sugar alcohols (xylitol), and choline derivates, DESs are called natural deep eutectic solvents (NADESs) [6,7,8,9]. In addition, NADESs may often contain water as a component at certain molar ratios [6]. Choi et al. [10] introduced the term NADES based on the hypothesis that these primary metabolites, present in high concentration in cells, may form a third type of liquid phase, apart from water and lipids. NADES may be involved in the biosynthesis, storage, and transport of several poorly water-soluble compounds in organisms. Choi et al. also showed that the solubility of the flavonoid rutin was from 50 to 100 times higher in NADESs than in water [10]. The considerable number of possible constituents stands as a main advantage of these solvents, allowing for a task-specific design of fluids for diverse technological applications [1,3,11,12]. The physicochemical properties of NADESs can be fine-tuned by modulating (i) their composition, both qualitatively and quantitatively, and (ii) the factors that affect their viscosity, including water content and temperature [3].

The high viscosity of many NADESs leads to some problems in their large-scale use, including time-consuming solvent transfer operations and slow mass transfer in extractions. This viscosity is related to the extensive network of weak bonding among components that decreases their mobility in the eutectic system [2,4]. NADES viscosity usually decreases when temperature is increased or when water is added in the range of 5–30%; this incorporation of water can be an integral part of NADES preparation and/or a diluting addition after preparation [2,6]. Considering that increasing temperature and/or diluting with water may disrupt the eutectic environment of NADESs, and that a wide variety of NADESs can be prepared, studies about how water affects the viscosity and the thermal stability of NADESs are relevant for their applications. This type of study generally relies on rheological and differential scanning calorimetry (DSC) measurements [6,13]. DESs and NADESs have been used to extract various flavonoid compounds, such as quercetin, kaempferol, and catechin from plant tissues, plant-based products, and biomass [14]. These DES extracts have exhibited comparable antioxidant properties, including the ability to scavenge DPPH and hydroxyl radicals, when compared to ethanol extracts [15].

Quinoa (*Chenopodium quinoa* Willd., Amaranthaceae) is rapidly gaining popularity as a functional food and nutraceutical around the world [16,17]. Quinoa seeds are a rich source of flavonoids, saponins, and phytoecdysteroids with a wide range of potential beneficial health effects, e.g., a reduction in risks for cardiovascular diseases, cancers, neurodegenerative diseases, diabetes, etc. [18,19,20,21]. Quinoa leaves, despite containing higher levels of phenolic compounds than seeds, remain underutilized and poorly explored in terms of their nutraceutical value [18,19]. Polyphenols, including flavonoids, are widely distributed plant phytochemicals known for their antioxidant properties, which include free radical scavenging, metal chelation, and redox activity. These compounds have been associated with the prevention of chronic diseases such as cardiovascular disorders, diabetes, and atherosclerosis. Recent in vitro studies have demonstrated the high bioavailability of phenolic compounds from quinoa leaves and their cytotoxic effects on prostate cancer cells, highlighting their potential role in cancer prevention and in mitigating oxidative stress-related conditions [18]. Our recent qualitative studies demonstrated that, compared to the widely praised quinoa seeds, quinoa leaves possess a high polyphenol content with strong free radical scavenging activity and notable α-amylase inhibitory properties, regardless of quinoa variety, phenological stage, or cultivation location [22,23].

The aim of the current study is to study eight types of NADESs (including some variations in water contents) for an eco-extraction of quinoa leaves bioactive compounds (flavonoids, free radical scavengers, and α-amylase inhibitors).

## 2. Results and Discussion

### 2.1. Preparation of Natural Deep Eutectic Solvents

The eight selected NADESs contain water as an integral component, which is reported to positively influence the stability of the eutectic mixture [5,6,9]; according to the reported molar ratios [5,6,9,24], they were prepared liquid mixtures by heating and stirring in the conditions described in Table 1. Choline chloride can form eutectic mixtures with various hydrogen bond donors (HBDs) [5], such as carboxylic acids (NADES N1) [9,24], sugars (NADES N2) [5,6], and polyols (NADES N5, N7, and N8) [5,6,9,25]. Other studies have shown that NADESs can also be formulated using carboxylic acids like malic acid and lactic acid in combination with amino acids or sugars (NADESs N3 and N6) [6,9,24,25]. Additionally, some NADES formulations are based entirely on mixtures of sugars (NADES N4) [6,9]. The present study tested these various possibilities on quinoa compounds and aimed at optimizing the viscosity of published NADES. As NADES N4 crystallized quite rapidly, some characterizations were not possible. The other eutectic mixtures were very stable at room temperature and did not undergo crystallization or phase separation during the tested times of storage (cf. Table 1).

This information is relevant for their eventual application in the extraction of natural compounds, which requires solvents that are sustainable and have long-term stability. Indeed, eutectic systems that tend to crystallize are not suitable for practical applications, as a key advantage of NADESs is their ability to preserve compounds extracted from natural sources—such as quinoa leaves—at room or refrigerated temperatures, while maintaining their structural integrity over extended periods (≥3 months) [8].

NADESs N1, N2, N3, and N4 were systems with notably high viscosity. Therefore, different proportions of dilution water (10%, 15%, 17.5%, 20%, and 50% *w*/*w*) were added to each eutectic mixture to reduce their viscosities.

### 2.2. Thermal Behavior of Prepared Natural Deep Eutectic Solvents

**Figure 1:**
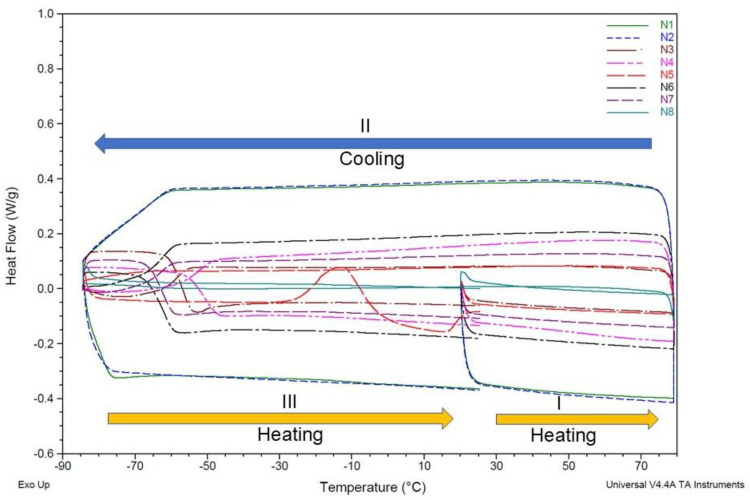
DSC Thermogram obtained at 10 °C/min for eight undiluted NADESs. Ramps: (I) heating from 30 °C to 80 °C; (II) cooling from 80 °C to −80 °C; and (III) heating from −80 °C to 30 °C. N5 exhibits exo and endo transitions due to crystallization and melting in the reheating scan.

The thermal behavior of the eight undiluted NADESs was analyzed using DSC just after their preparation. Figure 1 indicates that all NADESs have a glass transition point below −50 °C; no melting point could be detected in the thermograms. We observed no thermal phenomena within this range of temperature, showing that these eutectic mixtures present a liquid phase stability over this large range of temperatures [1,26], except for N5 (1,2 propanediol:choline chloride:water, molar ratio 1:1:1). This eutectic system had two thermal transitions during ramp III, one exothermic at −13 °C, followed by another endothermic at 13 °C, due to “cold” crystallization and melting, respectively; cold crystallization is crystallization undergone by an amorphous material upon reheating above its glass transition [27].

With the exception of NADES 5, the thermal stability of the evaluated NADESs indicates that, unlike conventional solvents (i.e., often toxic, volatile, and flammable solvents), these eutectic systems will not suffer from undesirable physical or chemical changes in the investigated temperature range, confirming that they are supramolecular complexes whose components interact via a sturdy network of intermolecular hydrogen bonds [9,27]. Dai et al. [9] reported a series of eutectic mixtures in which the incorporation of specific molar ratios of water in NADES preparation was compatible with the stability of liquid NADESs at room temperature, as confirmed by nuclear magnetic resonance (NMR) spectroscopy. These molar ratios were adopted in the present study. The same authors also reported that the water activity values of most DESs were approximately 0.2—significantly lower than the mole percentage of water in each system—indicating that the water is strongly bound within the matrix and not readily evaporated. This structural stability was further supported in our study using differential scanning calorimetry (DSC), which showed no thermal transitions in the thermograms of the eutectic mixtures, confirming the stability of the systems.

#### Effect of Water Addition on the Thermal Stability of Natural Deep Eutectic Solvents N1 to N4

The obvious high viscosity of NADESs N1 to N4 limits their applications in many fields, notably in extraction processes, due to the slowness of mass transfers. Diluting NADESs with water may be an effective mean to decrease their viscosity [15,28], but it may affect the eutectic environment of the NADES; it is then necessary to evaluate the effect of water addition on the thermal stability of the NADES.

**Figure 2:**
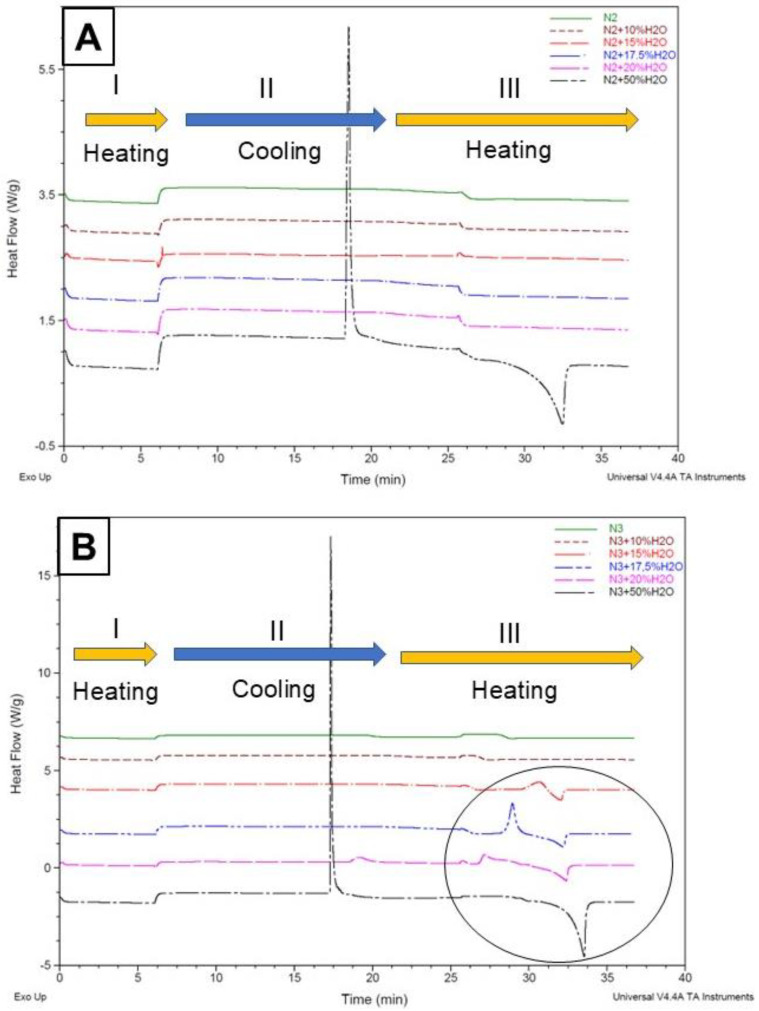
(**A**) Effect of water dilutions on the thermal stability of NADES N2. (**B**) Effect of water dilutions on the thermal stability of NADES N3. The solid circle indicates how increasing the water content in the dilution of the eutectic mixture leads to more pronounced transitions in the thermogram.

**Figure 3:**
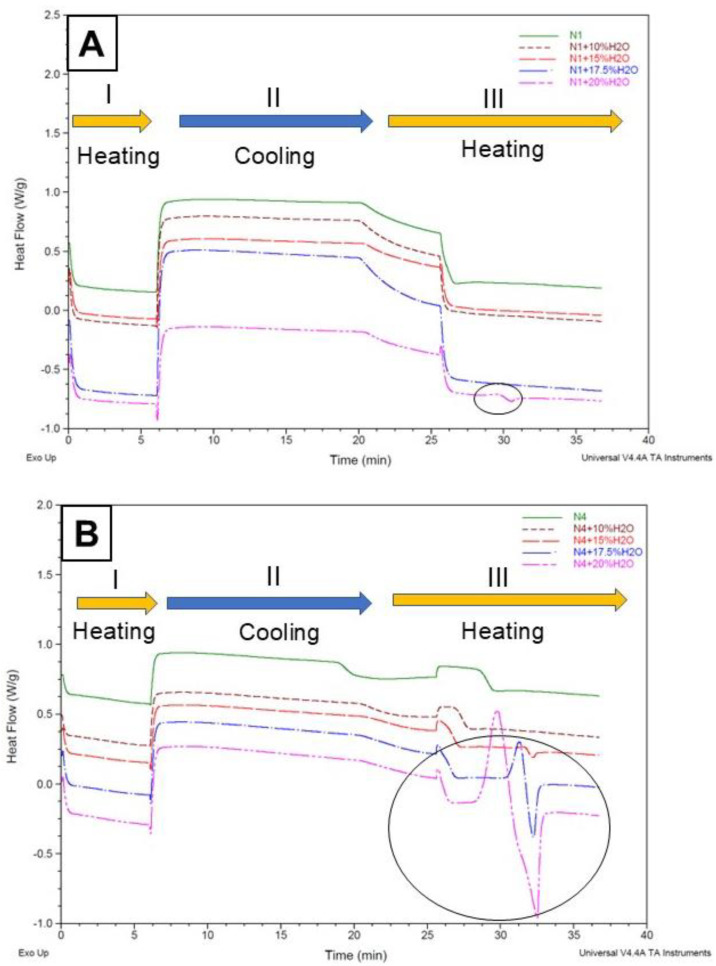
(**A**) Effect of water dilutions on the thermal stability of NADES N1. (**B**) Effect of water dilutions on the thermal stability of NADESs N4. The curves for 50% (*w*/*w*) of water are not included in order to observe the smaller transitions. The solid circle indicates how increasing the water content in the dilution of the eutectic mixture leads to more pronounced transitions in the thermogram.

The thermograms of N2 and N3 (Figure 2) and N1 and N4 (Figure 3), each diluted with increasing proportions of water, indicate that (i) the addition of water to NADESs N1, N3, and N4 promotes an increase in thermal transition areas; (ii) the stability of N2 is not affected by the addition of water up to 20% (*w*/*w*), as no thermal transitions are detected within the studied temperature range (Figure 2A); (iii) the eutectic nature of N3 (Figure 2B) is affected by the addition of 15% of water, exo, and endothermic peaks appearing in the thermograms; and (iv) when 50% (*w*/*w*) of water is added to N2 and N3, the exo and endothermic transitions are remarkably intense.

Dai et al. [6] examined how water dilution affects the structure and properties of various NADESs. They diluted a 1,2-propanediol-choline chloride-water eutectic mixture with deuterium oxide and analyzed it using NMR at 40 °C. The findings showed that the NADES’ structure remained intact only when the water content was below 50%. Beyond this point, the mixture was transformed into a solution containing the individual components dissolved in water. Hammond et al. [29] later analyzed the nanostructure of the DES system (choline chloride:urea:water) across a wide hydration range, revealing that low water levels slightly enhance hydrogen bonding between choline and urea. However, at 51% *w*/*w* of water, the DES structural motifs disappeared, and the system became an aqueous solution of the DES components. These data indicate that, upon excessive water addition, the unique solvation properties of NADESs disappear, virtually leading to extraction with a purely aqueous solvent.

In line with these studies, our data indicate that a weakening of between-components interactions, induced by an excess of water, that is both HBA and HBD, does affect the stability and structure of an eutectic system and increase the mobility of its components [6,27] to the point that our thermograms indicate a disappearance of interactions when diluting with water at 50% (*w*/*w*).

Figure 2 and Figure 3 indicate that the maximum amounts of water that can be added to preserve the eutectic properties of NADES N1, N2, N3, and N4 are 17.5, 20, 10, and 10% *w*/*w*, respectively. These dilutions were used for the extraction of bioactive compounds from quinoa leaves.

From these thermograms, DSC appears as an easy alternative to NMR to determine the maximum amount of water that can be added to an eutectic mixture to reduce its viscosity while preserving its supramolecular structure [26].

### 2.3. Rheological Measurements

**Figure 4:**
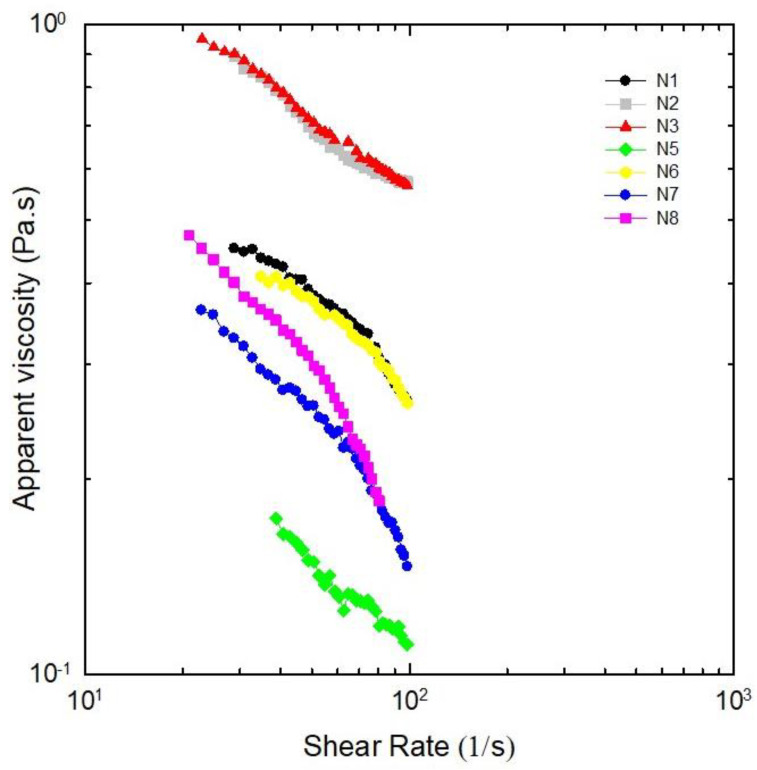
Log plots of viscosities as a function of shear rate for the studied NADESs (40 °C). N4 is not included because it crystallized before measurements.

Characterization of NADESs by their flow properties is relevant to ensure their applications in large-scale processes, because one of the major challenges regarding NADESs is to regulate their pumping costs and deal with their viscous behavior [3,6]. Rheological measurements, i.e., flow behaviors related to apparent viscosity [30] under varied shear rates, were performed after about two months of NADES preparation, except for N4, which crystallized in-between. The shear rate–shear stress curves were fitted to the power law model for the different NADESs studied in Figure 4; Table 2 presents the consistency coefficient (k), flow behavior index (*n*), and variation in apparent viscosity related to shear rate. For all dispersions, the viscosity decreased when the shear rate increased from 10 to 100 s^−1^, confirming pseudoplastic behavior.

The pseudoplastic behavior of NADESs are advantageous in extraction processes, as it facilitates an easier flow and mobility of flavonoids. It also aids in the mixing, dispersion, and dialysis of extracted compounds, resulting in higher extraction yields. The viscosity of pseudoplastic fluid decreases with shear stress generated by agitation during the extraction process, which enhances the transfer of flavonoids from quinoa leaves to the NADES, even at constant temperature [31]. Low-viscosity liquids are desirable for extraction processes. N1, N2, N3, and N6 appear to be unsuitable due to their high viscosities, a factor identified as one of the main limitations restricting the industrial application of these emerging solvents [2]. The development of low-viscosity solvents is then essential for broadening the practical applications of these novel green solvents [2,32]. NADES N5, N7, and N8 show potential for enhancing mass transfer in solid–liquid extractions, as they exhibit the lowest viscosity values. However, N5 and N8 are unstable, crystallizing in less than 6 months (cf. Table 1). In this context, NADES N7—characterized by both low viscosity and high stability, remaining a liquid mixture at room temperature for over 33 months—emerges as the most promising solvent for the extraction of bioactive compounds from quinoa leaves.

#### Effect of Water as Diluent on Viscosities of NADES N2

**Figure 5:**
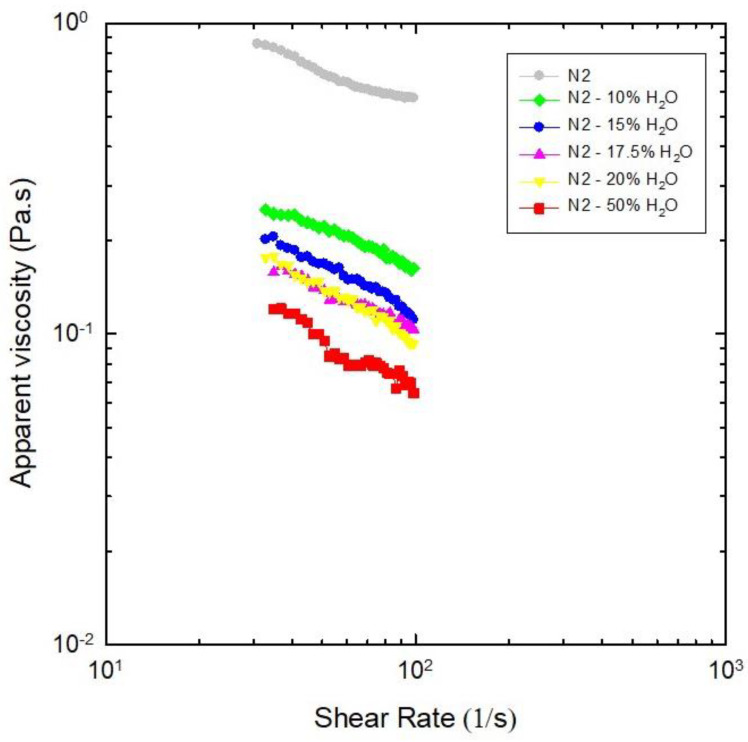
Log plots of viscosities as function of shear rate for N2 (choline chloride:glucose:water, molar ratio 5:2:5) diluted with different amounts of water (40 °C).

As N2 was the viscous NADES accepting the highest amounts of water, the effects of dilution on its viscosity were similarly evaluated; Figure 5 illustrates the significant decrease in viscosity of N2 with the addition of water, as expected [3,6,15]. The consistency coefficients (k) and flow behavior indexes (*n*) presented in Table 2 as a function of water content indicate that, for all dilutions of NADES N2, the viscosity decreased when the shear rate increased from 10 to 100 s^−1^. This general tendency confirms pseudoplastic behavior.

These data indicate that, when 10% (*w*/*w*) of water are added to N2 (choline chloride:glucose:water, molar ratio 5:2:5), the viscosity decreases by about 70%; further increases in water up to 20% induced smaller and smaller decreases in N2 viscosity, while the destruction of its eutectic properties by 50% of water had a more intense effect. These findings are consistent with those of Dai et al. [9], who reported that the viscosity of the same eutectic system decreases by 33% and 90% when diluted with 5% and 10% (*v*/*v*) of water, respectively.

### 2.4. HPTLC Fingerprints of Flavonoids in Recovered NADES Extracts

The extraction of bioactive compounds from quinoa leaves was performed using NADESs from N1 to N4 diluted with water (% *w*/*w*: N1—17.5%, N2—20%, N3—10%, and N4—10%) and NADESs from N5 to N8 undiluted.

**Figure 6:**
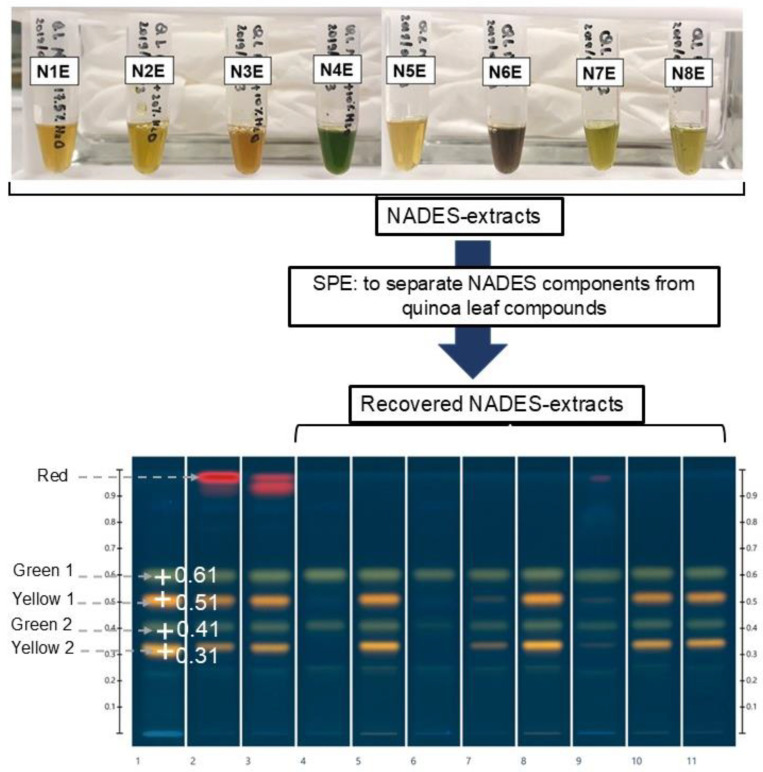
Colors and HPTLC flavonoid fingerprints of the eight NADES extracts (from N1E to N4E, NADES diluted with water as per Table 1; from N5E to N8E, undiluted NADES) obtained from quinoa leaves (sample/solvent ratio, 1:20 *w*/*w*; application volumes, 4 µL). Mobile phase: formic acid–water–methyl ethyl ketone–ethyl acetate (10:10:30:50, *v*/*v*/*v*/*v*). Derivatization with NP and PEG; examination under UV365 nm. Tracks: (1) aqueous extract; (2) methanolic extract; (3) methanol:water (80:20, *w*/*w*) extract; from (4) to (11) SPE recovered NADES extracts, from N1R to N8R.

Figure 6 shows that the colors of NADES extracts are different, depending on the NADES’ composition. Extracts N1E, N2E, N5E, N7E, and N8E were slightly yellow and less colored than N3E, N4E, and N6E. Less colored natural extracts are generally preferred, as they can be directly included in food and pharmaceutical products [26].

Direct application of NADES extracts on the HPTLC plate disturbed the chromatographic migration, the components of NADESs inducing severe tailing of spots in all samples, even when diluting 1/10 with water (Figure S1 in Supplementary Information). Therefore, as previously proposed [24], SPE was systematically applied to remove the NADES components, yielding “recovered NADES extracts” (cf. Section 3.5), which improved the quality of the chromatographic separations, preventing tailing.

**Figure 7:**
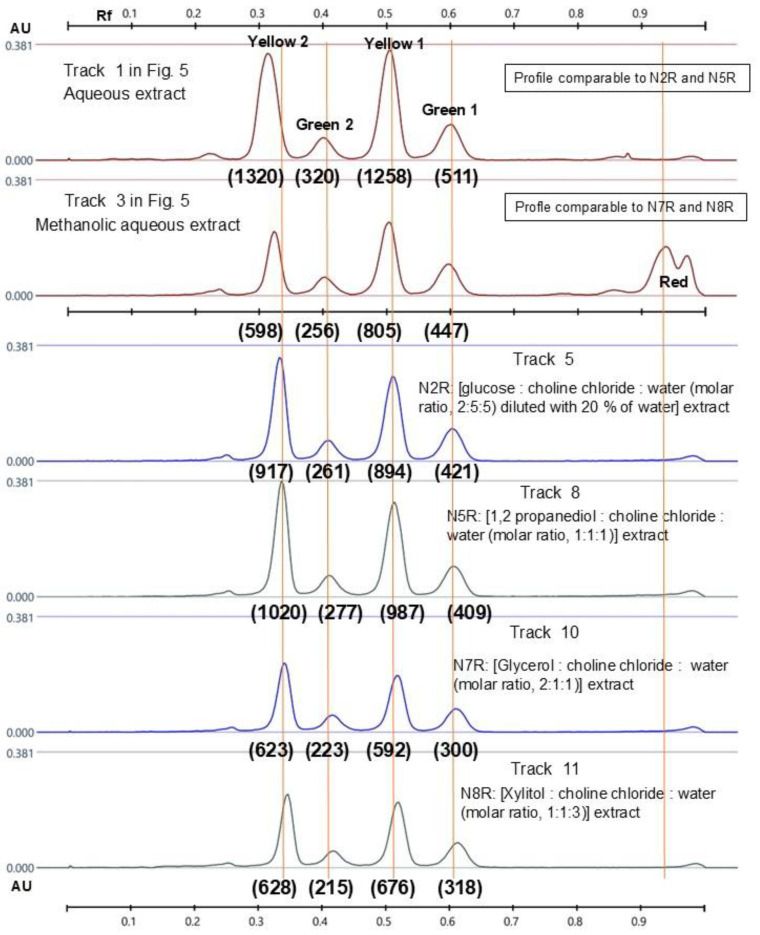
Peak profiles generated from Figure 6, i.e., the HPTLC plate examined under UV365 nm after derivatization with NP and PEG. Tracks: (1) aqueous extract; (3) methanol:water (80:20, *w*/*w*) extract; (5), (8), (10), and (11) SPE recovered NADES extracts N2R, N5R, N7R, and N8R.

Visual examination of the HPTLC chromatograms (Figure 6) indicates that both water and methanol:water (80:20, *w*/*w*) extract abundant amounts of quercetin (yellow spots) and kaempferol glycosides (green spots), with methanol being less efficient. Despite their efficiencies, these solvents pose problems; water promotes physical, chemical, and microbiological undesirable changes in the samples, and methanol is a toxic solvent and extracts chlorophylls (identified as red bands in tracks 2 and 3), yielding highly colored solutions. Some of the green solvents, N2 (diluted with 20% water), N5, N7, and N8 appear comparable to water and methanol:water for their efficiency in extracting flavonoids, which is confirmed by the integration of peak profiles (Figure 7), indicating that (i) N2R and N5R extracts are qualitatively and quantitatively similar in their polyphenol content to the aqueous extract, and that (ii) N7R and N8R are similar to the methanol:water extract. N5 and N8, however, were found to be unstable (Table 1), which would limit their use for industrial applications, and so the best candidates to replace water or methanol in the extractive processes of quinoa leaves would be N2 (choline chloride:glucose:water at molar ratio 5:2:5) (diluted with 20% water) and N7 (choline chloride:glycerol:water at molar ratio 1:2:1).

Our results with N7 reflect the finding of Fraige et al. [33], who showed, on *Byrsonima intermedia* leaves, that methanol:water (7:3 *v*/*v*) and a DES, based on choline chloride:glycerol at a molar ratio 1:2 diluted to 20% of water, both extract comparable amounts of quercetin glycosides (quercetin-O-hexoside, galloyl quercetin hexoside, quercetin-O-pentoside, and galloyl quercetin pentoside). Similar results were reported by Terrigno et al., who found that the total polyphenol content in *Eruca sativa* Mill. extracts were 2 mg GAE/g dw, regardless of the type of solvent used, including methanol and a eutectic mixture based on choline chloride-glycerol (molar ratio 1:2). Interestingly, the same authors reported that eutectic mixtures based on chloride-glucose extracted three times more total polyphenols than methanol [12]. This finding aligns with the results reported by Nam et al., who observed that the eutectic system based on L-proline-glycerol was tailored to provide greater extraction efficiency than methanol for the extraction of quercetin, kaempferol, and isorhamnetin glycosides from *Flos sophorae* [34].

Eutectic system based on choline chloride with HBD consisting of polyols, carboxylic acids, or amides are the most frequently reported for an efficient extraction of polyphenols, including flavonols [4,5,11,12,14]; polyols are particularly advantageous due to their hydroxyl groups, which form abundant hydrogen bonds with polyphenols, thereby enhancing their extraction. In fact, the eutectic system composed of choline chloride:glycerol:water (molar ratio 1:2:1) has demonstrated an enhanced extractability of saponins compared to conventional solvents [4,5,33,35,36].

HPTLC profiles of N1R, N3R, N4R, and N6R showed less intense and fewer bands than other extracts, but all of the main compounds appeared to be glycosides of kaempferol (green spots). This finding somewhat differs from that reported by Liu et al., who indicated that a NADES choline chloride:malic acid at molar ratio 1:1 diluted with 10% of water (i.e., ~3 times less water compared to N1) was the most efficient for the extraction of polyphenols from *Ginkgo biloba* leaves [24].

### 2.5. Free Radical Scavenging Capacity of Recovered NADES Extracts

**Figure 8:**
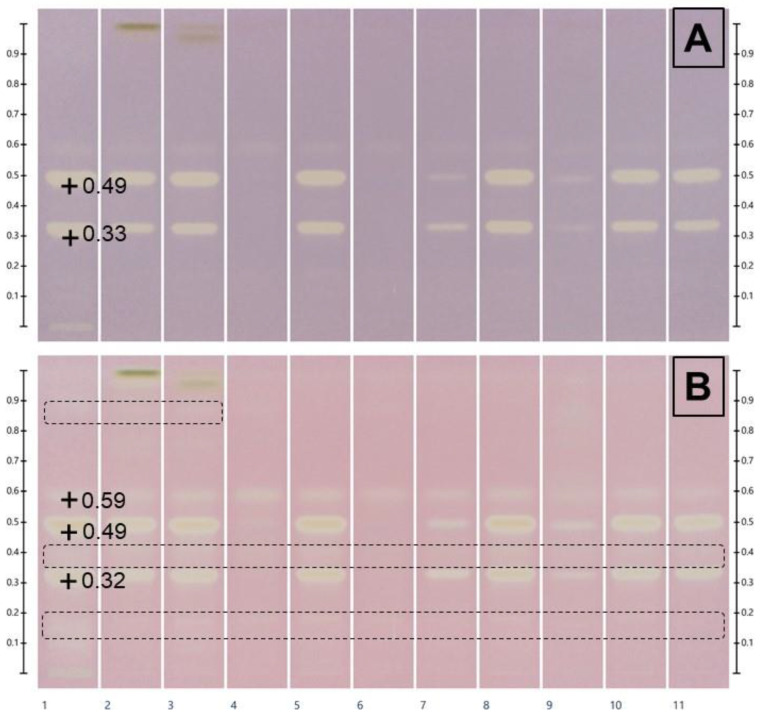
Free radical scavenging activity of the 8 NADES extracts (from N1E to N4E, NADESs diluted with water as per Table 1; from N5E to N8E, undiluted NADESs) obtained from quinoa leaves (sample/solvent ratio, 1:20 *w*/*w*; application volumes, 6 µL). Mobile phase: formic acid–water–methyl ethyl ketone–ethyl acetate (10:10:30:50, *v*/*v*/*v*/*v*). Derivatization with DPPH● for 90 s (**A**) and 120 min (**B**) and examination under visible light. Tracks: (1) aqueous extract; (2) methanolic extract; (3) methanol:water (80:20, *w*/*w*) extract; from (4) to (11) SPE recovered NADES extracts, from N1R to N8R. Low-intensity bands marked with a black dotted line.

The DPPH● assay, based on single-electron transfer mechanism, is a simple and common method for evaluating the free radical scavenging capacity of a sample that can be related to its antioxidant activity [37]. The quinoa leaves constituents contributing to DPPH● quenching can be separated and directly detected on HPTLC plates as yellowish spots on a purple background (Figure 8), with the distinct advantages of easiness and rapidity [38,39]. The intensity of reagent discoloration is proportional to a compound free radical scavenging potency and amount. Discoloration also depends on the reaction time, a complete reaction sometimes requiring from 30 to 60 min [38,39] and leading to a modification of the linearity domain [39]. Ninety seconds after derivatization, the same white zones (Rf 0.49 and 0.33) appear in all quinoa leaf SPE-recovered extracts, except for N1 and N3; these bands are less intense for N4 and N6. Upon maintaining the plate at RT in the dark for 120 min, the intensity of discolored zones increased, and new yellowish bands appeared (Rf 0.59 and low-intensity bands marked with a black dotted line).

When comparing the HPTLC profiles of flavonoids (Figure 6) and DPPH● quenchers (Figure 8B), the most intense quenchers (Rf 0.49 and 0.32) correspond to the yellow bands attributed to quercetin glycosides and only to one (Rf 0.59) of the 2 major kaempferol derivatives. Due to their radical scavenging properties, flavonoids are considered major contributors to the antioxidant activity of quinoa leaves [21]. In a comparative in vitro study, Tian et al. reported that luteolin, kaempferol, and quercetin exhibited stronger antioxidant activities than butylated hydroxytoluene. Moreover, luteolin and quercetin demonstrated superior antioxidant activity compared to vitamin C, as evidenced by the DPPH• radical scavenging assay, ABTS^+^• radical scavenging assay, and FRAP assay [40]. Structure–activity relationship studies indicate that the type and intensity of the antioxidant activity of flavonoids depend on the number and position of hydroxyl groups on the A and B rings, the possibility to oxidize in quinones, as well as the degree of conjugation between the B and C rings. These three main structural factors contribute to the effective free radical scavenging capacity of flavonoids [41].

But, indeed, previous studies [37,42] indicate that there is not necessarily a correlation between a polyphenol content and DPPH• scavenging, the quality of a given polyphenol/flavonoid being more important than its amount [15,42]. Nevertheless, for quinoa leaves, we found a clear correlation as the radical scavenging bands correspond to glycosides of quercetin and kaempferol.

### 2.6. α-Amylase Inhibitory Activity of Recovered NADES Extracts

Our four typical eutectic system based on their component types, i.e., N1 (base and acid), N2 (base and sugar), N3 (amino acid and acid), and N4 (sugar mixture), were selected for an initial screening of α-amylase inhibitors in quinoa leaves, applying a HPTLC bioautography method optimized in our laboratory [22].

**Figure 9:**
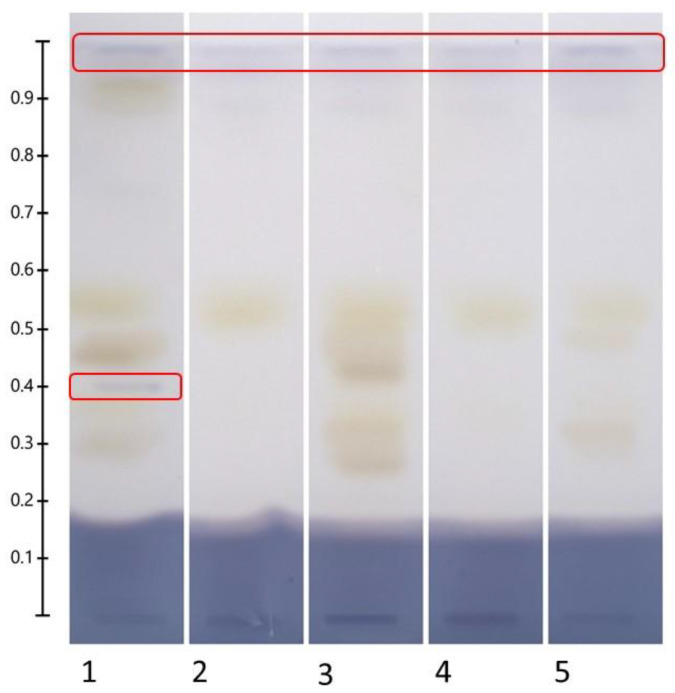
α-amylase inhibitory activity of leaf quinoa extracts (sample/solvent ratio, 1:20 *w*/*v*; application volumes, 8 µL). Mobile phase: formic acid–water–methyl ethyl ketone–ethyl acetate (10:10:30:50, *v*/*v*/*v*/*v*). Derivatization with α-amylase, starch, and iodine; examination under visible light. Tracks: (1) methanolic extract; from (2) to (5) SPE recovered NADES extracts, from N1R to N4R. The red marks indicate α-amylase inhibition zones. The large blue smear at the bottom of the plate (Rf from 0.0 to 0.2) is due to inhibition of α-amylase by the formic acid of the mobile phase.

A clear and defined blue zone (Figure 9; Rf 0.96, marked with a solid red line) was detected in all samples evaluated (methanolic, N1, N2, N3, and N4), regardless of the type of solvent, but a second blue area (Rf 0.4) was detected only in the methanolic extract. To the best of our knowledge, this study is the first in which NADESs are used for α-amylase inhibitors extraction; these results do not support the hypothesis that the α-amylase inhibitors detected using HPTLC-bioautography are phenolic compounds, as the Rf values do not concord with each other.

Quinoa leaves have seldom been investigated for such inhibitors, in contrast with the seeds, for which in vitro studies reported a strong inhibition of α-amylase and α-glucosidase activities [19,43]. Graf et al. reported that quinoa seeds possess in vivo anti-diabetic properties, attributed to leached phytoecdysteroids and flavonoids; these induce a significantly lowered fasting blood glucose in obese, hyperglycemic mice [16]. Many studies have shown that flavonoids exhibit not only well-recognized antidiabetic and hypoglycemic activities, but also activity in the treatment of diabetic complications [44]. It would be interesting to further investigate these activities for the NADES extracts of leaves.

## 3. Materials and Methods

### 3.1. Plant Material

Quinoa leaves of the INIAP-Tunkahuan variety were grown at the Instituto Nacional de Investigaciones Agropecuarias (INIAP), Santa Catalina Experimental Station, Pichincha province, 0°22′01″ S 78°33′17″ W, altitude 3050 m.a.s.l. Upon collection, the leaves were washed with distilled water, and the excess water was removed with a paper towel. The material was then lyophilized, ground, and stored at −20 °C. All material was ground in a laboratory mill (PX-MFC 90 D, Kinematica AG, Malters, Switzerland) and sieved to obtain particle sizes ≤ 0.5 mm.

### 3.2. Chemicals and Reagents

Choline chloride (≥98%), D-(+)-glucose (≥99.5%), 2,2-di (4-tert-octylphenyl)-1-picrylhydrazyl (DPPH●), 2-aminoethyl diphenylborinate (97%) (NP reagent), α-amylase from Bacillus licheniformis (Cat. No. A4582—5 mL), starch soluble (ACS reagent), and iodine (≥99.8%) were purchased from Sigma Aldrich; DL-malic acid (99%) and lactic acid (90% in aqueous solution), absolute ethanol (≥99.8%), polyethyleneglycol 400 (PEG 400), methanol (99%), methyl ethyl ketone (GPR Reactapur), formic acid (98%), and ethyl acetate (ACS reagent) from VWR; D-(-) fructose (≥99.5%), D-(+)-saccharose (≥99.5%) and L-proline (≥98.5%) from Roth; glycerol (99%) and methanol (99.8%) from Chem-Lab; 1,2-propanediol ACS reagent from Acros Organics; and xylitol 99% from Alfa Aesar. Water was Plus Millipore milli-Q grade (18.0 MΩ cm). Solid phase extraction (SPE) cartridges, StrataTM-X 33 µm, and Polymeric Sorbent were obtained from Phenomenex.

### 3.3. NADES Prepararion

Eight eutectic mixtures were prepared using a heating and stirring method [20]. Typical mixture components and molar ratios were selected from different NADES publications, based on their component types, i.e., NADESs composed of base and acid (N1), base and sugar (N2), amino acid and acid (N3), sugar mixtures (N4), and acid and sugar (N6) [9,24]. In addition to these typical eutectic systems, other NADESs were selected from their stated remarkable easiness of preparation and low viscosity, i.e., N5, N7, and N8, based on choline chloride and polyols; specifically, N7 (choline chloride: glycerol) was reported for the efficient extraction of *Byrsonima intermedia* A.Juss. leaf polyphenols [33]. The different components and conditions of preparation are described in Table 1.

### 3.4. Characterization of NADESs

#### 3.4.1. Thermal Stability

Differential scanning calorimetry (DSC) curves were recorded using DSC Q2000 equipment (TA Instrument Inc., Waters, New Castle, DE, USA) running under a nitrogen atmosphere, with 5–10 mg of sample packed in Tzero aluminum pans with hermetic lids. Experimental running was at a temperature ranging from −80 °C to 80 °C with 3 successive ramps: (I) heating from 30 to 80 °C; (II) cooling from 80 °C to −80 °C; and (III) heating from −80 °C to 30 °C. The heating rate was set at 10 °C/min [9].

#### 3.4.2. Rheological Measurements

Rheological characterization of NADESs was conducted using steady shear tests using a CVO Bohlin Instrument Rheometer with parallel-plate geometry and cone diameter of 20 mm. About 0.5 mL of the sample was placed on the lower base, which was set at 40 °C. Rheological tests were conducted at 40 °C, as numerous studies have indicated that solid–liquid solvent extractions of phenolic compounds from quinoa are typically performed at temperatures ranging from room temperature to 60 °C [15,19,45]. Exceeding this range may lead to considerable drawbacks, most notably the thermal degradation of the target compounds resulting from extended exposure to elevated temperatures [45]. The apparent viscosity–shear rate behavior of the NADES was determined by applying an increasing shear rate from 0.1 to 100 s^−1^, as reported in [30]. Three replicates of each sample were analyzed. The flow behavior index (*n*) and consistency index (k) values were computed by fitting to the Power Law model:

$$\tau =k{\dot{\gamma }}^{n}$$

where τ is the shear stress (Pa), $\dot{\gamma }$ is the shear rate (s^−1^), k is the consistency coefficient (Pa·s^n^), and *n* is the flow behavior index.

### 3.5. Preparation of Extracts and Sample Solutions for HPTLC Analysis

In total, 0.300 g of quinoa leaf powder was mixed with 6.000 g of a given NADES (diluted or not with water, cf. Table 1) in a 15 mL Falcon tube (sample/solvent ratio, 1:20 *w*/*w*), vortexed for 2 min, heated in a water bath at 40 °C for 1 h, sonicated at room temperature (RT) for 30 min, and centrifuged (4000× *g*, 40 min, 25 °C; UniCen MR, Herolab, Wiesloch, Germany) [24]. The supernatant (“NADES-extract”) was carefully separated from its solid phase using a Pasteur pipet. Solid-phase extraction (SPE—Strata X cartridges, Phenomenex, Torrance, CA, USA) was applied to recover the extract from the NADES components. The cartridge was placed on a vacuum manifold, equilibrated with 5 mL of ethanol and 5 mL of water, loaded with 1 mL of sample, rinsed twice with 6 mL of water, and then eluted with 6 mL of ethanol. The ethanol was dried, and the sample dissolved in 1 mL of methanol (“recovered NADES-extract”).

### 3.6. Characterization of Recovered NADES-Extracts

#### High-Performance Thin-Layer Chromatography

HPTLC was performed according to the general chapter 2.8.25 of the *European Pharmacopoeia 10* [46]. Fingerprinting of flavonoids was performed using HPTLC according to the procedure reported by Liu et al. [24]; antioxidant and α-amylase inhibitory zones were visualized using the protocol reported by Agatonovic-Kustrin et al. [38], with some modifications [22]. Samples were applied on HPTLC silica gel 60 F254 plates, size 20 × 10 cm (Merck, Darmstadt, Germany), with spray, using an Automatic TLC Sampler (ATS 4, Camag, Muttenz, Switzerland). Amounts of 4, 6, and 8 µL of the samples were sprayed onto the plate for the flavonoid detection protocol, the antioxidant activity assay (DPPH●), and the α-amylase inhibition assay, respectively; 15 bands of 8 mm were applied per plate, 8 mm from the plate’s lower edge; the plates were equilibrated under a 33% relative humidity and developed over a pathway of 70 mm from the lower edge in an Automated Multiple Development chamber (AMD2, Camag, Muttenz, Switzerland) with formic acid–water–methyl ethyl ketone–ethyl acetate (10:10:30:50, *v*/*v*/*v*/*v*). The plates were dried automatically for 5 min after development and heated at 105 °C for 60 min using a TLC Plate Heater (Camag, Muttenz, Switzerland). All reagents were sprayed with the Derivatizer Camag^®^ using the appropriate nozzle.

- Flavonoids: In total, 2 mL of aminoethyl diphenylborinate solution was applied on the warm plate (green nozzle, level 3) followed by 2 mL of polyethyleneglycol 400 solution (blue nozzle, level 2).
- Free radical scavenging activity (DPPH● assay): In total, 2 mL of DPPH solution was applied on the warm plate (green nozzle, level 3). Images were recorded 90 s and 120 min after derivatization.
- α-Amylase inhibitory activity assay: In total, 3 mL of α-amylase solution was applied (yellow nozzle, level 4) on the cooled plate that was then incubated at 37 °C for 30 min in a humid chamber [19]. Then, 2 mL of starch solution was applied (yellow nozzle, level 6), and the plate was incubated at 37 °C in a humid chamber for 10 min and treated with iodine vapors for 2 min (1 g of solid iodine in a 20 × 20 cm development chamber).
- Upon derivatization, the plates were documented as digital images under short-wave UV light (254 nm), long-wave UV light (365 nm), and white light using the TLC Visualizer 2. The Camag^®^ systems were driven using the visionCATS software, version 2.5.

## 4. Conclusions

Given the huge impact of human activity on the environment, the development of sustainable and green processes is encouraged and the green extraction of compounds from natural resources is set to become a crucial step in the development of dietary supplements or pharmaceutical drugs. In this sense, NADESs present the opportunity to develop eco-friendly extraction processes without compromising the biological efficacy of extracted material. The present work has shown that each NADES has its own extraction selectivity for the different quinoa leaf compounds. Eutectic mixtures based on choline chloride:glucose:water (molar ratio, 5:2:5) diluted with 20% of water and choline chloride:glycerol:water (molar ratio, 1:2:1) appear as potential candidates to replace methanol, water, and their mixtures in the extraction process for the eco-friendly extraction of flavonoids, radical scavengers, and α-amylase inhibitors from quinoa leaves. Their biological properties, combined with a good understanding of both thermal behavior and viscosity, make the obtained quinoa leaf NADES extracts good candidates for direct incorporation in nutraceutical formulations. The stability, bioavailability, and safety of these are pivotal to investigations of future pharmaceutical applications.

## Figures and Tables

**Table 1:** Components of natural deep eutectic solvents and their molar ratios: preparation temperature (T); preparation time (tp); storage time (ts) during which the undiluted eutectic mixtures remained stable at room temperature; and the proportion of diluted water to reduce the viscosity while maintaining an eutectic state.

NADES	Components for Preparation				Molar Ratio	T (°C)	tp ^(a)^ (min)	ts ^(b)^ (Months)	Proportion of Water to Dilute NADES Prior to Extraction Procedure ^(d)^
	1	2	3	4					
N1	Choline chloride	Malic acid	Water		1:1:2	50	440	>33 ^(c)^	17.5%
N2	Choline chloride	Glucose	Water		5:2:5	60	720	>33 ^(c)^	20%
N3	Proline	Malic acid	Water		1:1:3	50	360	>33 ^(c)^	10%
N4	Fructose	Glucose	Saccharose	Water	1:1:1:11	50	600	<2	10%
N5	Choline chloride	1,2 Propanediol	Water		1:1:1	50	30	<6	n.n. ^(e)^
N6	Glucose	Lactic acid	Water		1:5:3	60	60	>20 ^(c)^	n.n. ^(e)^
N7	Choline chloride	Glycerol	Water		1:2:1	50	20	>20 ^(c)^	n.n. ^(e)^
N8	Choline chloride	Xylitol	Water		2:1:3	50	60	<6	n.n. ^(e)^

^(a)^ tp: Preparation time of the eutectic mixture (in minutes). ^(b)^ ts: Storage time of the eutectic mixture at room temperature. ^(c)^ No crystallization or phase separation at the indicated storage time (i.e., NADESs considered to be stable). ^(d)^ Visually, N1, N2, N3, and N4 were the most viscous NADESs. To improve the extraction process, the proportion of dilution water appropriate for viscosity decrease without affecting the NADES’ eutectic environment were determined using DSC and rheology. ^(e)^ n.n.: water dilution was not needed because there was already a second liquid component aside from water to reduce the viscosity of the eutectic mixture.

**Table 2:** Fitting parameters to power law curves for NADES viscosity measurements (40 °C).

NADES	k (Pa·s^n^)	*n*	R
N1	2.2	0.55	0.9844
N2	3.0	0.63	0.9941
N3	3.1	0.63	0.9990
N4 ^(a)^	----	----	----
N5	0.8	0.57	0.9878
N6	2.0	0.57	0.9880
N7	2.5	0.41	0.9611
N8	3.9	0.33	0.9313

^(a)^ Not determined (crystallized before measurement). Flow behavior index (*n*) pseudoplastic nature (since *n* < 1) and consistency index (k) computed by fitting data of Figure 4 to the Power Law model $\tau =k{\dot{\gamma }}^{n}$, where τ is the shear stress (Pa), $\dot{\gamma }$ is the shear rate (s^−1^), and R is the correlation coefficient (n = 3 technical replicates).

## Data Availability

The original contributions presented in this study are included in the article/Supplementary Material. Further inquiries can be directed to the corresponding author.

## Supplementary Materials

The following supporting information can be downloaded at: https://www.mdpi.com/article/10.3390/molecules30122620/s1, Figure S1: High performance thin-layer chromatography plates (HPTLC) profiles of natural deep eutectic solvents-extracts, without solid phase extraction (SPE) treatment but diluted 1/10 (*v*/*v*) in water before application on the plate.
