# Supplementary material for: Natural Deep Eutectic Solvents (NADESs) for the Extraction of Bioactive Compounds from Quinoa (Chenopodium quinoa Willd.) Leaves: A Semi-Quantitative Analysis Using High Performance Thin-Layer Chromatography
Source: Molecules. 2025 Jun 17;30(12):2620. doi: 10.3390/molecules30122620 (PMC12195850; doi:10.3390/molecules30122620)
Supplement: Supplementary file 1 [file molecules-30-02620-s001.zip › molecules-3585188-supplementary.pdf]

Supplementary Information

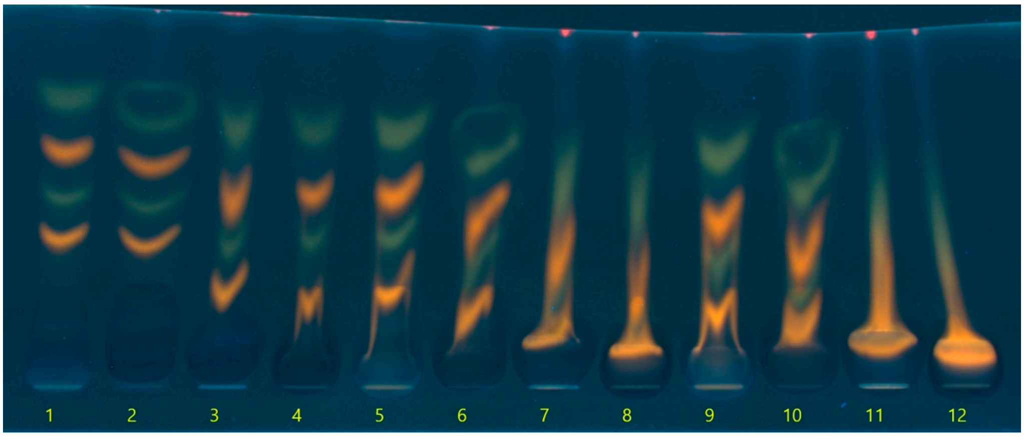

Figure S1. High performance thin-layer chromatography plates (HPTLC) profiles of natural deep eutectic solvents-extracts, without solid phase extraction (SPE) treatment but diluted 1/10 (v/v) in water before application on the plate. Samples:

- **N2 (diluted with 20% water, w/w): tracks 1 (20 µL), 5 (30 µL) and 9 (40 µL)**
- **N5: tracks 2 (20 µL), 6 (30 µL) and 10 (40 µL)**
- **N7: tracks 3 (20 µL), 7 (30 µL) and 11 (40 µL)**
- **N8: tracks 4 (20 µL), 8 (30 µL) and 12 (40 µL)**
